# Adiponectin Is a Component of the Inflammatory Cascade in Rheumatoid Arthritis

**DOI:** 10.3390/jcm11102740

**Published:** 2022-05-12

**Authors:** Małgorzata Łączna, Patrycja Kopytko, Marta Tkacz, Katarzyna Zgutka, Michał Czerewaty, Maciej Tarnowski, Dariusz Larysz, Rafał Tkacz, Daniel Kotrych, Katarzyna Piotrowska, Krzysztof Safranow, Karolina Łuczkowska, Bogusław Machaliński, Andrzej Pawlik

**Affiliations:** 1 Department of Physiology, Pomeranian Medical University in Szczecin, Powstańców Wlkp. 72, 70-111 Szczecin, Poland; malgorzata.lubecka@pum.edu.pl (M.Ł.); patrycja.kopytko@pum.edu.pl (P.K.); tkacz.mag@gmail.com (M.T.); katgrymula@o2.pl (K.Z.); michal.czerewaty@wp.pl (M.C.); maciejt@pum.edu.pl (M.T.); piot.kata@gmail.com (K.P.); 2 Department of Trauma and Orthopaedic Surgery, 109 Military Hospital, Piotra Skargi 9-11, 70-965 Szczecin, Poland; dariuszlarysz@hotmail.com (D.L.); rafatkacz@gmail.com (R.T.); 3 Department of Orthopaedics, Traumatology and Orthopaedic Oncology, Pomeranian Medical University, Unii Lubelskiej 1, 71-252 Szczecin, Poland; dkotrych@wp.pl; 4 Department of Biochemistry and Medical Chemistry, Pomeranian Medical University, 70-111 Szczecin, Poland; chrissaf@mp.pl; 5 Department of General Pathology, Pomeranian Medical University in Szczecin, Powstańców Wlkp. 72, 70-111 Szczecin, Poland; karolina.luczkowska@pum.edu.pl (K.Ł.); machalin@pum.edu.pl (B.M.)

**Keywords:** adiponectin, rheumatoid arthritis, bone marrow, synoviocytes, infrapatellar fat pad

## Abstract

Adiponectin is a secretory protein of adipocytes that plays an important role in pathological processes by participation in modulating the immune and inflammatory responses. The pro-inflammatory effect of adiponectin is observed in rheumatoid arthritis (RA). In this study, we examined adiponectin plasma levels and the expression of adiponectin in bone marrow tissue samples, synovium samples, and infrapatellar fat pad samples from patients with osteoarthritis (OA) and RA. Additionally we examined the expression of adiponectin receptors AdipoR1 and AdipoR2 in synovium samples and infrapatellar fat pad samples from patients with OA and RA. We also assessed the correlations between adiponectin plasma concentrations, adiponectin expression in bone marrow, synovium, infrapatellar fat pad, and plasma levels of selected cytokines. We found increased expression of adiponectin in synovium samples and infrapatellar fat pad samples from patients with RA as compared to patients with OA. There were no statistically significant differences of adiponectin plasma levels and adiponectin expression in bone marrow tissue samples between OA and RA patients. There were no differences in the expression of AdipoR1 and AdipoR2 at the mRNA level in synovial tissue and the infrapatellar fat pad between RA and OA patients. However, in immunohistochemical analysis in samples of the synovial membrane from RA patients, we observed very strong expression of adiponectin in intima cells, macrophages, and subintimal fibroblasts, such as synoviocytes, vs. strong expression in OA samples. Very strong expression of adiponectin was also noted in adipocytes of Hoffa’s fat pad of RA patients. Expression of AdipoR1 was stronger in RA tissue samples, while AdipoR2 expression was very similar in both RA and OA samples. Our results showed increased adiponectin expression in the synovial membrane and Hoffa’s pad in RA patients compared to that of OA patients. However, there were no differences in plasma adiponectin concentrations and its expression in bone marrow. The results suggest that adiponectin is a component of the inflammatory cascade that is present in RA. Pro-inflammatory factors enhance the expression of adiponectin, especially in joint tissues—the synovial membrane and Hoffa’s fat pad. In turn, adiponectin also increases the expression of further pro-inflammatory mediators.

## 1. Introduction

Adiponectin, also known as Acrp30, adipoQ, ApM1, and GBP28, is a 28–30 kDa secretory protein of adipocytes encoded by the ADIPOQ gene [1,2]. Structurally, it consists of a fibrous subunit at the nitrogen terminus and a globular subunit at the carboxyl terminus [3]. It belongs to the soluble collagen superfamily and is homologous to complement factor C1q and the tumor necrosis factor (TNF) family [4].

White adipose tissue is very metabolically active, secreting cytokines, enzymes, and peptide hormones called adipokines. One of the adipokines is adiponectin, which has long been of interest to scientists due to its dual influence on pathological processes. On the one hand, adiponectin has anti-inflammatory properties in metabolic diseases, while in inflammatory diseases of the joints, it appears to be a pro-inflammatory factor [3].

The main biological functions of adiponectin include energy homeostasis via the stimulation of fatty acid biosynthesis in skeletal muscles and the inhibition of gluconeogenesis in the liver [4,5]. Adiponectin exerts its biological effects by binding primarily with two receptors, namely adiponectin receptor 1 (AdipoR1), and adiponectin receptor 2 (AdipoR2), with specific distributions and characteristics. AdipoR1 is involved in the modulation of AMP kinase, while AdipoR2 is involved in the activation of peroxisome proliferator-activated receptor α (PPARα) in the process of fatty acid metabolism. However, it is known that adiponectin receptors are widely distributed and expressed in multiple tissues [6]. 

Adiponectin also plays a role in pathological processes by participating in modulating the immune and inflammatory responses. Over the years, studies have revealed that adiponectin presents with dual functions, namely anti-inflammatory and pro-inflammatory effects, and in this way contributes to the pathogenesis of many diseases [7,8,9,10]. Adiponectin exerts beneficial anti-inflammatory properties on the cardiovascular system, including on atherosclerosis and on metabolic disorders (such as obesity and insulin resistance). The pro-inflammatory effects of adiponectin are observed in rheumatoid arthritis (RA), chronic kidney disease (CKD), and inflammatory bowel disease [11,12,13,14,15]. It is likely that both the anti-inflammatory and pro-inflammatory effects of adiponectin depend on the duration of exposure to this adipokine, its concentration, the tissues involved, and environmental factors such as other cytokines and the tissue microenvironment. Baker et al. suggest that adiponectin levels in RA patients did not depend on the body mass. Increased adiponectin levels may be associated with both excess adiposity and low lean mass in patients with RA [16]. 

Previous studies indicate that adiponectin can play both pro-inflammatory and anti-inflammatory roles in the pathogenesis of RA. In the joints, adiponectin stimulates the secretion of pro-inflammatory mediators increasing the inflammatory status [15]. These differences may be related to the ability of adiponectin to form multimeric complexes with different biological activities and the binding of adiponectin to their receptors with different affinities. It was shown that the AdipoR1 receptor binds the adiponectin trimer with higher affinity, while AdipoR2 shows a greater affinity to multimers [5].

To date, the precise role of adiponectin in RA has not been established. It is not known whether the observed elevated adiponectin levels in RA patients are the primary cause of the development of inflammation or whether they are a consequence of existing inflammation in these patients. It is not known in which tissues adiponectin plays a pro-inflammatory role in RA patients. It is unknown which mediators associated with inflammation in RA enhance adiponectin expression. It has been shown that RA patients have inflammatory changes in many organs, including the bone marrow [17,18]. 

In this study, we examined the expression of adiponectin in bone marrow, plasma, synovial membrane, and Hoffa’s fat pad, which is infrapatellar white adipose tissue, to evaluate in which tissues adiponectin is involved in the inflammatory process in RA. We also correlated plasma levels of adiponectin with plasma concentrations of selected pro-inflammatory mediators (IL-1β, IL-2, IL-4, IL-6, IL-7, IL-8, IL-10, IL-12 p40, IL-13, IL-17, G-CSF, GM-CSF, IFN-γ, MCP-1, MIP-1β, TNF-α) involved in the pathogenesis of RA to assess which mediators may influence adiponectin expression. 

In addition, we investigated the effects of inflammatory mediators (LPS, TNF) on adiponectin expression in human fibroblast-like synoviocytes (HFLSs) to assess whether inflammation in RA enhances adiponectin expression in affected tissues.

We also evaluated the effects of adiponectin on the expression of IL-6, a cytokine involved in the pathogenesis of RA.

Additionally, we examined the expression of adiponectin receptors AdipoR1 and AdipoR2 at the level of mRNA and in the immunohistochemical analysis in synovium samples and infrapatellar fat pad samples from patients with osteoarthritis (OA) and rheumatoid arthritis (RA).

## 2. Material and Methods

### 2.1. Synovial and Infrapatellar Fat Pad Samples

The study included 17 patients with rheumatoid arthritis (15 female, 2 male, mean age 60.5 ± 5.3 years) and 25 with osteoarthritis (20 female, 5 male, mean age 65.3 ± 7.8 years) who were undergoing joint replacement surgery as a normal part of clinical care. Clinical characteristics of RA and OA patients are shown in Table 1. OA patients were used as the control for the RA patients because of the different pathogenesis of these two diseases. The patients with diabetes, hypertension, or other conditions that might alter adiponectin concentrations were excluded from the study. Plasma adiponectin concentrations were additionally measured in 30 healthy subjects. 

During surgery, blood, bone marrow, synovial membrane, and fat pad samples were collected from each patient. Tissues, transported to the laboratory in a sterile saline solution, were processed within 2 to 6 h after removal from the patient. Immediately upon arrival, the tissues were washed with PBS (EURx) and finely minced into small pieces, transferred to a 50 mL Falcon tube, and digested with a solution of collagenase I (1 mg/mL) and collagenase II (0.5 mg/mL) (Sigma) with PBS for 90–120 min in 37 °C. The tissues were then filtered using a sterile 70 µm Falcon cell strainer. Cells were centrifuged in warm PBS (37 °C) for 10 min at 1800 rpm at room temperature (RT). Cell pellets were then resuspended in 1 mL of PBS (EURx) and transferred to a 1.5 mL tube. Cells were centrifuged for 5 min at 1500 rpm at RT. After centrifugation, the supernatants were discarded, and cell pellets were resuspended in 350 µL of RLT lysis buffer (Qiagen, Hilden, Germany). The cell samples were then stored at −20 °C for further RNA isolation procedures. The study was approved by the ethics committee at Pomeranian Medical University, Szczecin, Poland (KB-0012/39/17), and written informed consent was obtained from all subjects.

### 2.2. Bone Marrow Tissue Samples

Samples of bone marrow were obtained from patients with RA and OA who were undergoing knee synovectomy or joint replacement surgery as a normal part of clinical care. Tissues, transported to the laboratory in a sterile saline solution, were processed within 2 to 6 h after removal from the patient. Immediately upon arrival, the tissues were centrifuged for 10 min at 1800 rpm at RT. Bone marrow pellets were then resuspended in 10–15 mL of lysing buffer (BD, Franklin Lakes, NJ, USA) and incubated for 10 min at RT. The samples were then resuspended in PBS and centrifuged for 10 min at 1800 rpm at RT. After centrifugation, the supernatants were discarded, and cell pellets were resuspended in 350 µL of RLT lysis buffer (Qiagen). The cell samples were then stored at −20 °C for further RNA isolation procedures.

### 2.3. RNA Isolation

Total RNA was isolated from the synovial cells using an RNeasy^®^ Mini Kit (Qiagen), according to the manufacturer’s protocol.

### 2.4. RT-PCR

Isolated messenger RNA was reverse-transcribed using a cDNA synthesis kit (RevertAid RT Kit, Thermo Scientific, Waltham, MA, USA), according to the manufacturer’s protocol.

### 2.5. Real-Time Quantitative Reverse Transcription PCR (qRT-PCR)

Quantitative expression analysis of the selected genes, as well as the reference gene, was performed using real-time qRT-PCR on an ABI PRISM^®^ Fast 7500 Sequence Detection System (Applied Biosystems, Waltham, MA, USA). Real-time conditions were as follows: 95 °C (15 s), 40 cycles at 95 °C (15 s) and 60 °C (1 min). According to the melting point analysis, only one PCR product was amplified under these conditions. Each sample was analyzed in two technical replicates, and mean Ct values were used for further analysis. To calculate the values, the 2^−ΔCt^ method was used. The values were normalized to β2-microglobulin.

### 2.6. The Assessment of Cytokines in Plasma of Patients with RA and OA

The sixteen cytokines were tested in each sample by a magnetic bead based multiplex assay according to the manufacturer’s procedure (Bio-Plex Pro Human Cytokine 16-plex, Bio-Rad, Hercules, CA, USA): interleukin (IL)-1β, IL-2, IL-4, IL-6, IL-7, IL-8, IL-10, IL-12 p40, IL-13, IL-17, granulocyte colony-stimulating factor (G-CSF), granulocyte-macrophage colony-stimulating factor (GM-CSF), interferon-gamma (IFN-γ), monocyte chemotactic protein (MCP)-1, macrophage inflammatory protein (MIP)-1β, and tumor necrosis factor-alpha (TNF-α).

### 2.7. The Assessment of Adiponectin Concentration in Plasma of Patients with RA and OA

The adiponectin concentrations in plasma were tested in each sample by a magnetic bead based multiplex assay according to the manufacturer’s procedure (Luminex Assay R&D, Minneapolis, MN, USA).

### 2.8. Immunohistochemical (IHC) Localization of Adiponectin and Adiponectin Receptor 1 and Receptor 2 (AdipoR1 and R2) in Joint Tissues

Sections of knee joint tissues (synovial membrane and Hoffa’s fat pad) (5 μm thick) were hydrated, and heat epitope retrieval was performed in a microwave oven in citrate buffer at pH = 6 (Dako Retrieval Solution, Dako Denmark, Glostrup, Denmark). After cooling to room temperature (RT), tissues were attained with antibodies and visualized with a DAB chromogen-ImmPRESS^®^ HRP Universal (Horse Anti-Mouse/Rabbit IgG) PLUS Polymer Kit, Peroxidase, Vector Laboratories, Burlingame, CA, USA, according to the manufacturer’s protocol. Briefly, the activity of peroxidase was blocked with BLOXALL Endogenous Enzyme Blocking Solution (ImmPRESS^®^ HRP Universal (Horse Anti-Mouse/Rabbit IgG) PLUS Polymer Kit, Peroxidase, Vector Laboratories, Burlingame, CA, USA), washed twice with PBS, and further incubated with 2.5% Normal Horse Serum (ImmPRESS^®^ HRP Universal PLUS Polymer Kit, Peroxidase, Vector Laboratories, USA). Furthermore, slides were incubated with primary monoclonal antibodies, namely anti-adiponectin, anti-AdipoR1, and anti-AdipoR2 (SantaCruz Biotechnology, Dallas, TX, USA) for 1 h in RT. After a double wash in PBS, slides were incubated with ImmPRESS Universal Antibody Polymer Reagent (ImmPRESS^®^ HRP Universal PLUS Polymer Kit, Peroxidase, Vector Laboratories, USA), and after washing in PBS, the reaction was visualized with ImmPACT DAB EqV Substrate(ImmPRESS^®^ HRP Universal PLUS Polymer Kit, Peroxidase, Vector Laboratories, USA). For the negative control, primary antibodies were replaced with PBS. After visualization, slides were counterstained with hematoxilin (Harris modified Hematoxilin, Sigma, St. Louis, MO, USA) and mounted in Canada balsam (Sigma-Aldrich, Satin-Louis, MI, USA) mounting medium and evaluated under an Olympus IX81 inverted microscope (Olympus, Hamburg, Germany). Micrographs were collected with CellSens software (Olympus, Germany).

### 2.9. Immunoreactive Score Estimation for Adiponectin and Adiponectin Receptors R1 and R2

Immunoreactive scores (IRSs) for adiponectin and AdipoR1 and AdipoR2 on synovial membranes and fat pads samples were calculated as described elsewhere [19]. Briefly, each patient’s sample was evaluated in 5 random chosen fields of view (×40 objective). The immunoreactive score was calculated as IRS = estimated staining intensity (color reaction intensity 0–1–2–3–) multiplied by the estimated proportion of cells with positive reactions (0–1–2–3–4), where 0 is 0%, 1 is 1–11%, 2 is 11–50%, 3 is 51–80%, and 4 is 81% to 100% positive stained cells. Values of IRS: 0–12, where 0 is no reaction, and 12 is a very strong reaction in 81% to 100% cells, described as 0—negative, 1 to 6—weakly positive, and 7 to 12—strongly positive [19]. Data are shown in the table as mean, minimal, and maximal value for IRS [20]. The procedure of IRS estimation was conducted by two independent rounds for each slide for each patient from the RA (n = 10) and OA (n = 10) groups.

### 2.10. Fibroblast-Like Synoviocyte Cultures

Human fibroblast-like synoviocytes (HFLSs) were purchased from Cell Applications, Inc. (San Diego, CA, USA). Cells were cultured in the HFLS Growth Medium all-in-one ready-to-use kit (Cell Applications, Inc., San Diego, CA, USA) under an atmosphere of 5% CO_2_ in air (*v*/*v*) at 37 °C at an initial cell density of 2.5 × 10^4^ cells/flask (Corning, New York, NY, USA). The medium was replaced every 2–3 days.

### 2.11. Cell Culture Stimulation

Cells were harvested from the culture flasks by trypsinization, and the number of cells was determined using a Bürker hemocytometer (Buffalo, NY, USA). Cells were seeded in 24-well plates at a density of 0.07 × 106 cells per well and incubated for 24 h with HFLS Growth Medium all-in-one ready-to-use kits (Cell Applications, Inc., San Diego, CA, USA). Afterwards, the medium was replaced by HFLS Basal Medium (Cell Applications, Inc., San Diego, CA, USA) containing low levels of bovine serum albumin (BSA; 0.5%) (Sigma) to render the cells quiescent for 24 h. The cells were washed with PBS (EURx) the next day, and stimulating factors were added.

#### 2.11.1. TNF and LPS Stimulation

The cells were stimulated for 6, 12, 24, and 72 h with tumor necrosis factor-α (TNF-α) (10 ng/mL), lipopolysaccharide (LPS) (100 ng/mL), or both. HFLS Basal Medium with BSA 0.5% (Sigma) was used as a negative control. The samples were performed in triplicate. After the time points, the cell pellets were used for total RNA isolation and reverse transcriptase-polymerase chain reaction (RT-PCR) analysis.

#### 2.11.2. LPS and Adiponectin Stimulation

The cells were stimulated for 24 and 72 h with optimal doses of LPS (Sigma) and adiponectin (Peprotech, Inc., Cranbury, NJ, USA), respectively, in different variants: (1) LPS1000 ng/mL, (2) adiponectin 250 ng/mL, (3) adiponectin 1000 ng/mL, (4) adiponectin 250 ng/mL + LPS1000 ng/mL, and (5) adiponectin 1000 ng/mL + LPS1000 ng/mL. HFLS Basal Medium with BSA 0.5% (Sigma) was used as a negative control. The samples were performed in triplicate. After the time points, the cell pellets were used for total RNA isolation and reverse transcriptase-polymerase chain reaction (RT-PCR) analysis. LPS was derived from *E. coli* bacteria (lipopolysaccharides from *Escherichia coli* O55: B5) and was purchased from Sigma-Aldrich, St. Louis, MO, USA (Cat # L6529).

### 2.12. Statistical Analysis

Since distributions of quantitative variables significantly differed from a normal distribution (Shapiro–Wilk test), non-parametric tests were used. Values were compared between groups with Kruskal–Wallis or Mann–Whitney tests, and correlations within groups were assessed with Spearman rank correlation coefficients. ANOVA with a Tukey post hoc test was used to analyze quantitative expression results, which were transformed logarithmically to normalize their distributions. For the analysis of adiponectin expression, the independent variables were: presence of one of the four possible combinations of stimulating factors (control—no stimulation factor, TNF only, LPS only, TNF + LPS) and time of stimulation (6, 12, 24, or 48 h). For the analysis of IL-6 expression, the independent variables were: presence of one of three concentrations of adiponectin (0, 250, or 1000 ng/mL), presence of LPS (yes or no), and time of stimulation (24 or 72 h). *p* < 0.05 was considered statistically significant.

## 3. Results

### 3.1. Adiponectin Expression in Plasma, Bone Marrow, Synovium, and Infrapatellar Fat Pad Samples

The first point of our study was to see whether changes in adiponectin expression in RA patients occurred already in the bone marrow and plasma or only within the inflamed joint tissues. Therefore, we determined adiponectin levels in RA and OA patients in bone marrow tissue samples, plasma, the joint synovial membrane, and the infrapatellar fat pad.

**Figure 1:**
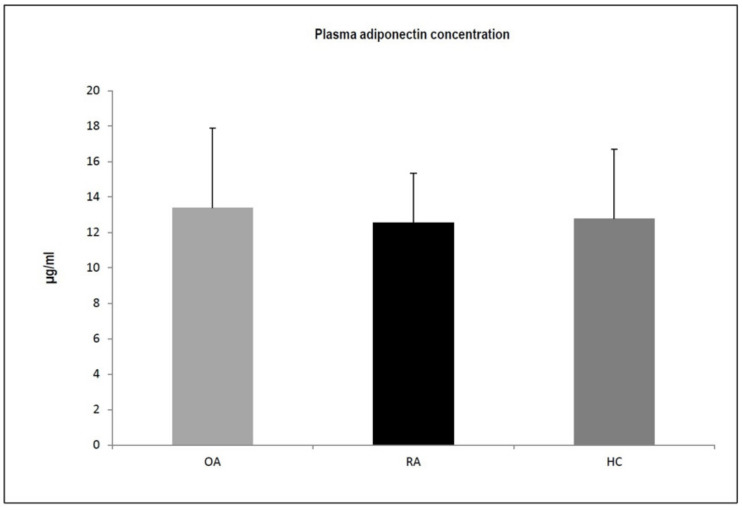
Plasma adiponectin concentrations at the protein level (mean ± SD, µg/mL) in OA—osteoarthritis and RA—rheumatoid arthritis patients and in healthy subjects (HC—healthy controls) (Kruskal–Wallis test).

**Figure 2:**
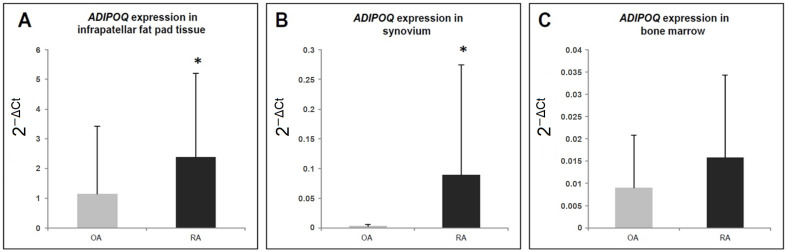
mRNA expression levels of adiponectin (*ADIPOQ*) in OA—osteoarthritis and RA—rheumatoid arthritis patients (mean ± SD): Panel (**A**) infrapatellar fat pad tissue, Panel (**B**) synovium, Panel (**C**) bone marrow. * *p* < 0.05 indicates a significant increase in gene expression in RA patients as compared to OA patients (Mann–Whitney test).

We compared adiponectin plasma concentrations at the protein level (Figure 1) and adiponectin mRNA expression in bone marrow tissue samples, synovium samples, and infrapatellar fat pad samples from patients with OA and RA (Figure 2). As shown in Figure 1, there were no statistically significant differences in adiponectin plasma levels between RA patients, OA patients, and healthy control subjects (*p* = 0.96, Kruskal–Wallis test). 

As shown in Figure 2C, there were no statistically significant differences in adiponectin expression at the mRNA level in bone marrow tissue samples between patients with OA and RA.

We observed the increased expression of adiponectin in synovium samples and in infrapatellar fat pad samples from patients with RA in comparison to patients with OA (Figure 2A,B).

### 3.2. Correlations between Plasma Concentrations of Adiponectin and Selected Pro-Inflammatory Cytokines

In patients with RA and OA, there are several interrelationships and feedback loops that regulate and exacerbate the synthesis of multiple pro-inflammatory mediators. Therefore, we investigated the correlations between plasma concentrations of selected pro-inflammatory cytokines involved in RA and OA pathogenesis and adiponectin plasma levels.

We examined the correlations between adiponectin plasma levels and plasma levels of selected cytokines (IL-1β, IL-2, IL-4, IL-6, IL-7, IL-8, IL-10, IL-12 p40, IL-17, G-CSF, GM-CSF, IFN-γ, MCP-1, MIP-1β, TNF-α) in patients with OA and RA.

In patients with OA, adiponectin plasma levels correlated significantly with plasma levels of IL-6. 

There were no statistically significant correlations between plasma concentrations of adiponectin and plasma concentrations of the studied cytokines (Table 2).

### 3.3. Assessment of mRNA Expression and Localization of Adiponectin and Adiponectin Receptor 1 and Receptor 2 (AdipoR1 and R2) in Joint Tissues

**Figure 3:**
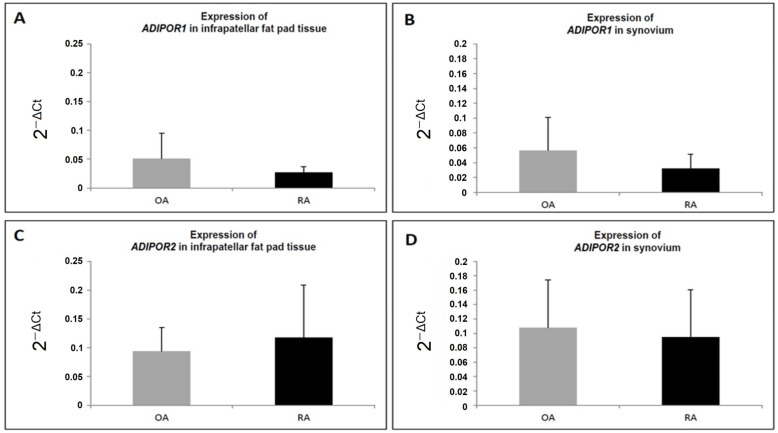
mRNA expression levels of adiponectin receptor 1 (ADIPOR1) and adiponectin receptor 2 (ADIPOR2) in OA—osteoarthritis and RA—rheumatoid arthritis patients (mean ± SD): Panel (**A**,**C**) infrapatellar fat pad tissue, Panel (**B**,**D**) synovium. There were no significant differences in ADIPOR1 and ADIPOR2 gene expression in RA patients as compared to OA patients (Mann–Whitney test).

The next point of our work was to investigate the expression and localization of adiponectin receptors in joint tissues. We examined the mRNA expression of AdipoR1 and R2 in synovial membrane and infrapatellar fat pad samples from patients with OA and RA. As shown in Figure 3, there were no statistically significant differences of AdipoR1 and R2 expression in synovial membrane and infrapatellar fat pad samples between patients with OA and RA.

We also assessed the localization of adiponectin and adiponectin receptor 1 and receptor 2 (AdipoR1 and R2) in joint tissues.

### 3.4. Adiponectin

**Figure 4:**
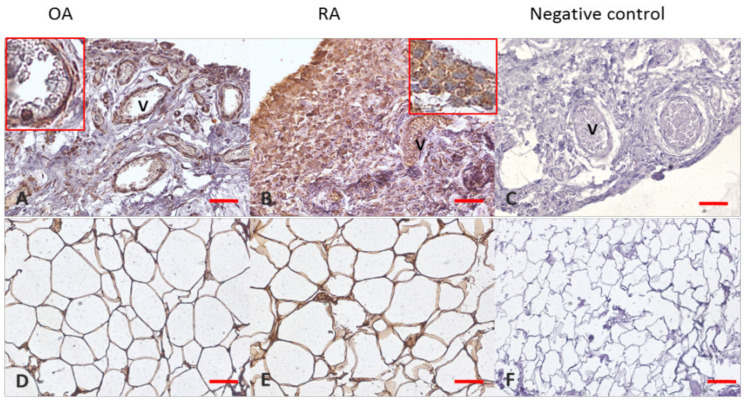
Adiponectin expression in joint’s tissues. Panels (**A**–**C**) synovial membranes; Panels (**D**–**F**) Hoffa’s fat pad. OA—osteoarthritis; RA—rheumatoid arthritis; V—vessel; Panels (**C**,**F**) show negative control staining. Objective magnification ×20, scale bar 50 μm; only representative images are presented. Insets show large magnifications of cytoplasmic staining in synovial membrane cells (Panel (**B**)) and endothelial cells (Panel (**A**)).

In knee joint tissues, we observed a strong (++) positive reaction in OA synovial intima cells (Figure 4A) and subintimal synovial fibroblasts and macrophages, and a strong (++) reaction was also observed in epithelial cells of vessels (marked as V). A strong positive reaction was noted in Hoffa’s fat pad in OA patients (Figure 4D). In samples from RA patients, we observed very strong (+++) expression of adiponectin in intima cells, macrophages, and subintimal fibroblasts; like synoviocytes (Figure 4B), a very strong reaction was also noted in adipocytes of Hoffa’s fat pad of RA patients (Figure 4E).

### 3.5. AdipoR1 and AdipoR2

**Figure 5:**
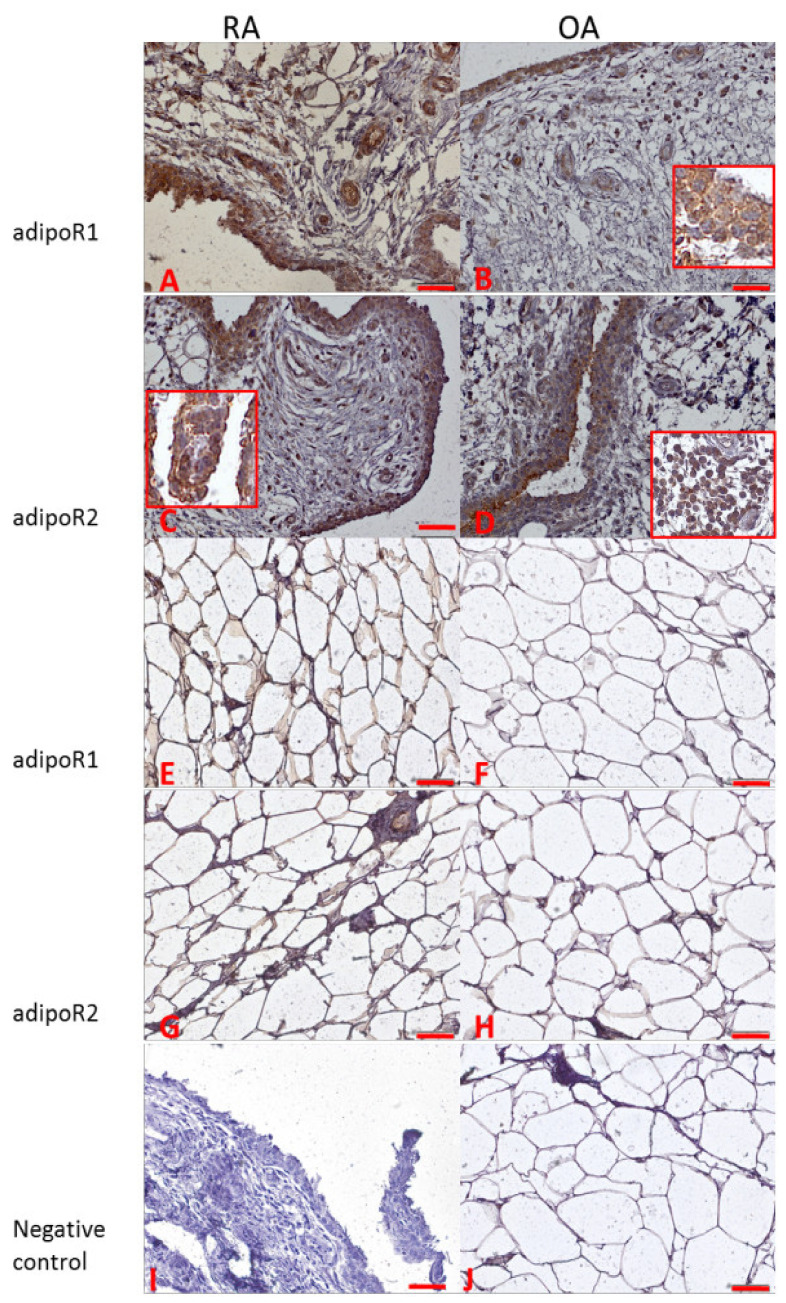
AdipoR1 and AdipoR2 expressions in joint tissues: Panels (**A**–**D**) synovial membranes; Panels (**E**–**H**) Hoffa’s fat pads. OA—osteoarthritis; RA—rheumatoid arthritis; Panels (**I**,**J**) show negative control staining. Insert shows gathered inflammatory cells positively stained for AdipoR2. Objective magnification ×20, scale bar 50 μm; only representative images are presented. Inserts show large magnification of cytoplasmic staining in synovial membrane cells—Panels (**B**,**C**), and immune cells staining—Panel (**D**).

In RA patients’ synovial membranes, we noted very strong (+++) cytoplasmic expression for AdipoR1 and strong expression for AdipoR2 (Figure 5A,C). In synovial membranes from OA patients, we observed strong (++) expression for AdipoR1 and AdipoR2 (Figure 5B,D). In Hoffa’s fat pad, a strong reaction for AdipoR1 was observed in RA samples (Figure 5E). In OA sections of Hoffa’s fat pad, we noted moderate expression (+) for AdipoR1 and AdipoR2 (Figure 5F,H). A similar moderate expression for AdipoR2 was observed in Hoffa’s pad in RA patients (Figure 5G). A positive reaction was noted in intima cells and synoviocyte-like fibroblasts, but positive staining was also visible in inflammatory cells dispersed or gathered in the subintimal region of the synovial membrane (Figure 5D insert).

Additionally, we performed immunoreactive score (ISR) estimation for adiponectin and adiponectin receptors R1 and R2.

The results of IRS estimation are presented in Table 3 and Table 4. The data are presented as mean values of the IRS and minimal and maximal values for each antibody in each tissue in each group of patients.

In the synovial membrane, the mean of IRS for adiponectin was strongly positive in the RA and OA groups (9 and 8), for AdipoR1 it was strongly positive in the RA group and weakly positive in the OA group (8 vs.6), and for AdipoR2 it was weakly positive in the RA and OA groups (6 vs.4).

In the fat pad, the IRS for adiponectin in the RA and OA was strongly positive (8), and AdipoR1 and AdipoR2 were weakly positive in RA and OA (4 and 2). In the case of AdipoR2, the mean value of IRS in the RA group was higher than in the OA group (4 vs. 2). Larger differences between minimal and maximal values for IRS in a given group show large individual differences between the patients within the group.

### 3.6. Stimulation of Human Fibroblast-Like Synoviocytes (HFLSs) with LPS and TNF-α

To assess whether pro-inflammatory mediators associated with RA development increase adiponectin expression in tissues, we stimulated the human fibroblast-like synoviocytes (HFLSs) with lipopolysaccharide (LPS) and tumor necrosis factor-α (TNF-α) for 48 h. TNF is one of the most important cytokines involved in the pathogenesis of RA, which increases inflammation, while LPS is a non-specific stimulator that increases inflammation. To best reflect the inflammation present in RA patients, cells were stimulated with LPS, because in RA, there are many inflammatory factors and mediators that exacerbate inflammation.

**Figure 6:**
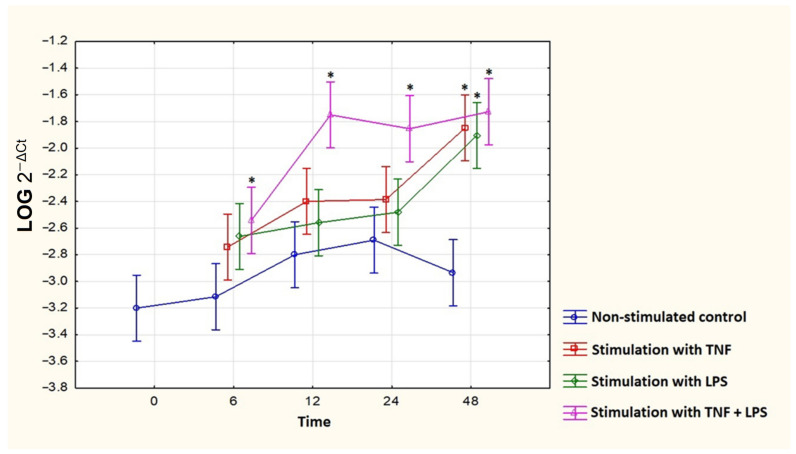
Effect of stimulation of human fibroblast-like synoviocytes (HFLSs) with LPS, TNF-α, and LPS + TNF-α on adiponectin mRNA expression. Means from 3 experiments ± 95% confidence intervals are presented. * *p* < 0.05 (Tukey post hoc test in comparison to non-stimulated control). TNF: Tumor necrosis factor. LPS: Lipopolysaccharide.

Stimulation with LPS and TNF-α alone resulted in a statistically significant increase in adiponectin mRNA expression in cultures of HFLSs after 48 h of stimulation. The stimulation with LPS plus TNF-α resulted in a statistically significant increase in adiponectin mRNA expression in cultures of HFLSs after 6, 12, 24, and 48 h of stimulation (Figure 6).

### 3.7. The Effect of Adiponectin on IL-6 mRNA Expression in Human Fibroblast-Like Synoviocytes (HFLSs)

**Figure 7:**
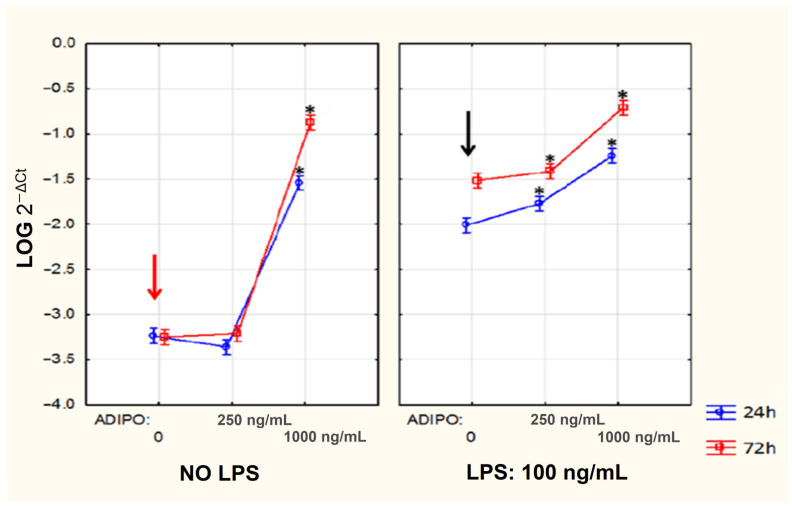
The effect of adiponectin (ADIPO) stimulation of human fibroblast-like synoviocytes (HFLSs) on *IL-6* mRNA expression. Red arrow indicates negative control (without adiponectin and without LPS). Black arrow indicates control with LPS alone (without adiponectin). Means from 3 experiments ± 95% confidence intervals are presented. * *p* < 0.05 (Tukey post hoc test in comparison to cells not stimulated with adiponectin).

Additionally, we examined the effect of adiponectin on the mRNA expression of *IL-6* in HFLSs. Figure 7 presents the relative expression of IL-6 in HFLS cultures after 24 and 72 h of stimulation with adiponectin. The cells were stimulated with adiponectin in concentrations of 250 ng/mL or 1000 ng/mL alone or together with LPS. The HFLSs were stimulated with LPS to investigate how adiponectin affects *IL-6* expression in the inflammatory environment present in RA.

Adiponectin alone in concentrations pf 250 ng/mL had no statistically significant effect on mRNA expression of IL-6. Adiponectin in concentrations of 250 ng/mL together with LPS caused statistically significant increases of IL-6 mRNA expression in cultures after 24 and 72 h of stimulation. Adiponectin in concentrations of 1000 ng/mL alone and together with LPS caused statistically significant increases of mRNA IL-6 expression in HFLS cultures after 24 and 72 h of stimulation (Figure 7).

## 4. Discussion

In this study, we investigated the plasma concentrations of adiponectin and the expression of adiponectin in bone marrow tissue samples, synovium samples, and infrapatellar fat pad samples from patients with OA and RA to determine whether changes in adiponectin expression in RA patients had occurred already in the bone marrow and plasma or only within the inflamed joint tissues. Additionally, we examined the correlations between adiponectin plasma levels with plasma levels of selected cytokines in patients with OA and RA to assess which pro-inflammatory mediators may influence adiponectin expression.

There were no statistically significant differences in adiponectin plasma levels and adiponectin expression in bone marrow tissue samples between patients with OA and RA. Adiponectin expression was statistically significantly increased in synovium samples and infrapatellar fat pad samples from patients with RA compared to patients with OA. 

We also examined the effect of mediators of inflammation (LPS, TNF-α) on the adiponectin expression in human fibroblast-like synoviocytes. Stimulation with LPS and TNF-α resulted in a statistically significant increase in adiponectin expression in cultures of HFLSs. These results indicate that inflammation in RA increases the expression of adiponectin. 

We also examined the effects of adiponectin on the expression of IL-6 in HFLS cultures. Adiponectin alone in concentrations of 250 ng/mL did not affect the expression of IL-6. However, adiponectin together with LPS, which is a known stimulator of inflammation, increased IL-6 expression. Adiponectin at 1000 ng/mL alone and together with LPS increased significantly IL-6 expression. These results indicate that adiponectin at low concentrations without inflammation did not increase IL-6 expression. In contrast, when inflammation was present, already low concentrations of adiponectin enhanced IL-6 expression. High levels of adiponectin even without the presence of inflammation enhanced IL-6 expression. These results may suggest that the action of adiponectin depends on its concentration and the microenvironment in which it acts. The presence of inflammation in RA patients may enhance the pro-inflammatory effects of adiponectin. Previous studies have shown that in many diseases where the inflammation is not observed, adiponectin exerts beneficial effects [11].

The role of adiponectin in the etiology and pathogenesis of RA has been tested in many studies [12,13,14,15]. Previous studies suggest the involvement of adiponectin in RA pathogenesis. Nevertheless, the results of the studies are often conflicting. The studies indicate both pro-inflammatory and anti-inflammatory roles of adiponectin in RA. The anti-inflammatory and pro-inflammatory effects of adiponectin appear to depend on the tissues on which it acts and on environmental factors such as other cytokines and the tissue microenvironment. The complex and multiple roles of adiponectin include immune regulation and alteration of the microenvironment through regulation of angiogenesis. Some studies reported increased plasma levels of adiponectin in RA patients, which correlated positively with the parameters of disease activity, clinical symptoms, and progression of joint erosions and also correlated with the expression of other mediators involved in inflammation, severity of joint destruction, and response to treatment [15,21,22,23,24]. Other studies have not found elevated plasma adiponectin concentrations in RA patients [25,26], or even a negative correlation between adiponectin concentrations and disease activity parameters [27,28].

Studies have shown that adiponectin plays a pro-inflammatory role in RA, increasing the secretion of other pro-inflammatory mediators by activating synovial fibroblasts. In vitro stimulated synovial fibroblasts produced several pro-inflammatory mediators, such as matrix metalloproteinases, IL-6, IL-8, and prostaglandin E2 [29,30,31,32]. However other studies indicated that in a mouse model of collagen-induced arthritis, adiponectin significantly reduced the severity of arthritis along with decreasing the expression of TNF-α, IL-1, and MMP-3 in joint tissues [33]. Adiponectin also inhibited synovial fibroblast proliferation induced by IL-1β, while increasing IL-6 expression in IL-1β-stimulated fibroblasts [34,35]. IL-6 seems to be a key pleiotropic factor in the development of chronic inflammation in RA. The result of the pleiotropic effects of IL-6 in the synovium include chronic synovitis, which causes proliferation of fibroblast-like synoviocytes (FLSs), angiogenesis, and degradation of the extracellular matrix of cartilage [14,15,36,37]. IL-6 is also involved in the activation of Tfh lymphocytes, whose main function is to regulate the differentiation of B cells in germinal centers into plasma cells and memory B cells [38].

Adiponectin may also modify the physiological functions of bone tissue cells. Osteoblasts stimulated in vitro by adiponectin had a reduced ability to mineralize the matrix. Adiponectin also stimulated osteoclasts to increase bone resorption, and it could also inhibit the expression of osterix (Osx), an osteoblast-specific transcription factor that is essential for bone formation. It also interferes with the inhibition of osteoclast activity by stimulating the expression of osteoprotogerin mRNA, thus delaying bone formation [5,39].

Recent studies have shown that adiponectin exhibits several another pro-inflammatory effects in RA. Huang et al. showed that adiponectin promotes VEGF expression in rheumatoid arthritis synovial fibroblasts and induces endothelial progenitor cell angiogenesis by inhibiting miR-106a-5p [40]. Adiponectin enhances B-cell proliferation and differentiation via activation of Akt1/STAT3 and exacerbates collagen-induced arthritis [41]. Recombinant adiponectin induces the production of pro-inflammatory chemokines, cytokines in circulating mononuclear cells, and fibroblast-like synoviocytes from non-inflamed subjects [42].

Our study showed no statistically significant difference in adiponectin plasma levels and adiponectin expression in bone marrow tissue samples between RA and OA patients. We have shown increased adiponectin expression in synovial samples and infrapatellar fat pad samples from patients with RA compared to patients with OA. Previous studies have shown both increased plasma adiponectin levels in RA patients and no increase in adiponectin levels in this patient group [15,21,22,23,25,27]. It probably depends on the activity and duration of the disease and comorbidities. It can be hypothesized that initially adiponectin concentrations in RA patients are not elevated, but in the tissues affected by the inflammatory process, under the influence of many pro-inflammatory mediators, adiponectin synthesis is increased. This may be suggested by the increased expression of adiponectin in the synovial membrane and infrapatellar fat pad. Moreover, our results showed that the stimulation of human fibroblast-like synoviocytes by TNF and LPS increased the expression of adiponectin. 

In addition, studies suggest that in newly-diagnosed RA, adiponectin exerts mainly anti-inflammatory effects, but pro-inflammatory effects of adiponectin are observed in patients with advanced disease. Chen et al. have shown a negative correlation between plasma adiponectin levels and disease activity parameters in newly diagnosed RA patients [43], while in studies including patients with advanced disease, a positive correlation of adiponectin with disease process activity parameters was observed [22]. 

Additionally, we examined the correlations between adiponectin plasma levels and plasma levels of selected cytokines in patients with OA and RA. In patients with OA, adiponectin plasma levels correlated significantly with plasma levels of IL-6. There were no statistically significant correlations between plasma concentrations of adiponectin and plasma concentrations of the studied cytokines. Vasileiadis et al. showed an association between circulating adiponectin and pro-inflammatory chemokines involved in RA pathogenesis, as well as markers of inflammation in patients with untreated newly diagnosed RA [44]. Our study included RA patients with long-standing, advanced disease. This may be a likely reason for the lack of correlation between adiponectin levels and other pro-inflammatory mediators. 

AdipoR1 signaling participates in the process of synovial inflammation and joint damage in collagen-induced arthritis [45]. In our study, we also examined the expression of adiponectin receptor 1 (AdipoR1) and receptor 2 (AdipoR2) on the level of mRNA and localization of AdipoR1 and AdipoR2 in joint tissues. There were no statistically significant differences in mRNA expression for AdipoR1 and AdipoR2 in joint tissues between RA and OA patients. However, in immunohistochemical analysis in samples of synovial membrane from RA patients, we observed very strong expression of adiponectin in intima cells, macrophages, and subintimal fibroblasts, such as synoviocytes. Very strong expression of adiponectin was also noted in adipocytes of Hoffa’s fat pad of RA patients. The expression of adiponectin was also detected in samples from OA patients. In knee joint tissues, we observed strong expression of adiponectin in OA synovial intima cells, subintimal synovial fibroblasts, and macrophages, as well as in epithelial cells of vessels. 

For estimation of differences in immunohistochemical detection of adiponectin and its receptors (AdipoR1 and AdipoR2), we used a standard scoring procedure, IRS, which is widely used for protein expression estimation with the IHC method on histopathological slides [19,20]. Our results showed differences in adiponectin and AdipoR1 and R2 expression in synovial membranes of RA patients in comparison to OA patients and in the fat pad for AdipoR2. No differences in expression were observed for adiponectin and AdipoR1 in fat pads for tested groups. 

The differences in adiponectin and their receptor expressions in tissues of RA and OA patients may be a result of different inflammatory status in RA and OA patients (strong inflammation in RA vs. weak inflammation in OA patients), and post-transcriptional modifications. It is likely that various pro-inflammatory mediators involved in the development of inflammation in the joint tissues of RA patients post-transcriptionally increase the expression of adiponectin receptors. This is consistent with the results of Tan et al., where differences in AdipoR1 and AdipoR2 expression were suggested to be a result of blood vessel pathologies [46]. In our study, we also noted high levels of expression of adiponectin receptors in blood vessel intimae.

A limitation of our study is the lack of measurement of tissue adiponectin concentrations at the protein level. However, our results indicate that the action of adiponectin in RA patients depends on the tissue, concentration, and microenvironment in which it acts, as well as the presence of other pro-inflammatory factors. Our results indicate that the increase in adiponectin expression occurs mainly in joint tissues, which may be affected by inflammation. This is probably due to stimulation by other pro-inflammatory mediators. On the other hand, adiponectin may also enhance the expression of some pro-inflammatory mediators.

## 5. Conclusions

Our results showed increased adiponectin expression in the synovial membrane and Hoffa’s pad in RA patients compared to OA patients. However, there were no differences in plasma adiponectin concentrations and its expression in bone marrow. In addition, we showed that pro-inflammatory mediators enhance the expression of adiponectin in fibroblast-like synoviocytes. On the other hand, adiponectin, especially in the presence of other inflammatory mediators, increased the expression of IL-6.

Our results suggest that adiponectin is a component of the inflammatory cascade in RA. Pro-inflammatory factors enhance the expression of adiponectin, especially in joint tissues—the synovial membrane and Hoffa’s fat pad. In turn, adiponectin also increases the expression of further pro-inflammatory mediators.

## Figures and Tables

**Table 1:** Clinical characteristics of patients.

	RA Patients	OA Patients
Male/female	15/2	20/5
Age (years)	60.5 ± 5.3	65.3 ± 7.8
Disease duration (years)	11.5 ± 3.2	13.7 ± 2.7
CRP (mg/dL)	25.4 ± 4.3	4.7 ± 1.5
WBC (G/L)	7.9 ± 2.1	6.8 ± 1.8
RBC (T/L)	4.1 ± 1.2	4.9 ± 1.9
Hb (g/dL)	12.8 ± 3.5	14.3 ± 3.7

CRP: C-reactive protein, WBC: white blood cells, RBC: red blood cell, Hb: Hemoglobin.

**Table 2:** Correlations between adiponectin plasma levels and selected cytokines (IL-1β, IL-2, IL-4, IL-6, IL-7, IL-8, IL-10, IL-12 p40, IL-17, G-CSF, GM-CSF, IFN-γ, MCP-1, MIP-1β, TNF-α) in OA and RA patients. Rs—Spearman rank correlation coefficient; G-CSF—granulocyte colony-stimulating factor; GM-CSF—granulocyte-macrophage colony-stimulating factor; IFN-γ—interferon-gamma; MCP-1—monocyte chemotactic protein; MIP-1β—macrophage inflammatory protein; TNF-α—tumor necrosis factor-alpha.

Parameters	Plasma Adiponectin Concentration			
	Osteoarthritis		Rheumatoid Arthritis	
	Rs	*p*	Rs	*p*
IL-1β	0.52	0.06	0.13	0.66
IL-2	−0.45	0.11	−0.03	0.91
IL-4	−0.37	0.2	0.01	0.96
IL-6	0.65	0.01	0.24	0.41
IL-7	−0.11	0.72	−0.01	0.97
IL-8	−0.52	0.06	0.03	0.92
IL-10	−0.33	0.25	−0.04	0.88
IL-12	−0.43	0.12	0.31	0.27
IL-17	−0.09	0.75	0.25	0.4
G-CSF	0.41	0.15	0.17	0.57
GM-CSF	0.37	0.2	0.06	0.84
IFN-γ	−0.37	0.19	0.05	0.85
MCP-1	−0.43	0.12	0.23	0.43
MIP-1β	−0.28	0.33	0.03	0.91
TNF-α	0.24	0.42	0.49	0.08

**Table 3:** Immunoreactive scores (IRS) for synovial membrane. IRS values: 0—negative, 1–6—weakly positive, 7–12—strongly positive.

Rheumatoid Arthritis			
Protein	Mean	Minimal Value	Maximal Value
Adiponectin	9	8	12
AdipoR1	8	2	12
AdipoR2	6	2	12
**Osteoarthritis**			
**Protein**	**Mean**	**Minimal Value**	**Maximal Value**
Adiponectin	8	2	12
AdipoR1	6	3	9
AdipoR2	4	0	9

IRS: immunoreactive score. OA: osteoarthritis. RA: rheumatoid arthritis.

**Table 4:** Immunoreactive scores (IRS) for fat pad. IRS values: 0—negative, 1–6—weakly positive, 7–12—strongly positive.

Rheumatoid Arthritis			
Protein	Mean	Minimal Value	Maximal Value
Adiponectin	8	4	12
AdipoR1	4	2	8
AdipoR2	4	2	8
**Osteoarthritis**			
**Protein**	**Mean**	**Minimal Value**	**Maximal Value**
Adiponectin	8	8	8
AdipoR1	4	0	6
AdipoR2	2	0	6

IRS: immunoreactive score. OA: osteoarthritis. RA: rheumatoid arthritis.

## Data Availability

Not applicable.
